# Effects of 4.9 GHz Radiofrequency Field Exposure on Brain Metabolomic and Proteomic Characterization in Mice

**DOI:** 10.3390/biology13100806

**Published:** 2024-10-10

**Authors:** Xing Wang, Guiqiang Zhou, Jiajin Lin, Zhaowen Zhang, Tongzhou Qin, Ling Guo, Haonan Wang, Zhifei Huang, Guirong Ding

**Affiliations:** 1 Department of Radiation Protection Medicine, School of Military Preventive Medicine, Fourth Military Medical University, Xi’an 710032, China; wxing1106@163.com (X.W.); zgqiang0308@163.com (G.Z.); linjiajin913@126.com (J.L.); zzw13781108770@163.com (Z.Z.); qtz832697@163.com (T.Q.); guo2016@fmmu.edu.cn (L.G.); whn0825@163.com (H.W.); hzf9803@163.com (Z.H.); 2 Ministry of Education Key Lab of Hazard Assessment and Control in Special Operational Environment, Xi’an 710032, China; 3 School of Public Health, Shandong Second Medical University, Weifang 261053, China

**Keywords:** radiofrequency field, metabolomic, proteomic, brain, serum

## Abstract

**Simple Summary:**

The brain, as the central nervous system that controls the body’s sensory, behavior, and mental symptoms, is sensitive to RF exposure, and lots of studies have explored the potential health hazards of RF-EMR with different frequencies to the brain. Our previous study found that 4.9 GHz radiofrequency radiation induced depression-like behavior in mice, but the mechanism of the behavioral changes was unclear. Studies have shown that changes in peripheral energy metabolism might affect brain lipid levels, and thereby cortical excitability, and a deregulated hippocampus proteome might influence the healthy functioning of the brain. Here, we provide evidence that 4.9 GHz RF exposure altered metabolite expression patterns in brain tissue and serum, especially glycerophospholipid metabolism. In addition, 4.9 GHz RF exposure induced an imbalance in the protein profile of brain tissue and may alter gap junction communication. Our results initially revealed the biological effects of 5G communication frequency exposure and provided a possible mechanism for electromagnetic radiation-induced behavioral changes from the perspective of metabolome and proteome.

**Abstract:**

Electromagnetic exposure has become increasingly widespread, and its biological effects have received extensive attention. The purpose of this study was to explore changes in the metabolism profile of the brain and serum and to identify differentially expressed proteins in the brain after exposure to the 4.9 GHz radiofrequency (RF) field. C57BL/6 mice were randomly divided into a Sham group and an RF group, which were sham-exposed and continuously exposed to a 4.9 RF field for 35 d, 1 h/d, at an average power density (PD) of 50 W/m^2^. After exposure, untargeted metabolomics and Tandem Mass Tags (TMT) quantitative proteomics were performed. We found 104 and 153 up- and down-regulated differentially expressed metabolites (DEMs) in the RF_Brain group and RF_Serum group, and the DEMs were significantly enriched in glycerophospholipid metabolism. Moreover, 10 up-regulated and 51 down-regulated differentially expressed proteins (DEPs) were discovered in the RF group. Functional correlation analysis showed that most DEMs and DEPs showed a significant correlation. These results suggested that 4.9 GHz exposure induced disturbance of metabolism in the brain and serum, and caused deregulation of proteins in the brain.

## 1. Introduction

Electromagnetic exposure is ubiquitous in modern social life, such as wireless communication, WiFi, and mobile phone base stations [1]. People are living in electromagnetic environments all the time, so it is crucial to explore the effects of electromagnetic exposure. Radiofrequency electromagnetic field (RF-EMF) is electromagnetic exposure with frequencies ranging from 100 kHz to 300 GHz [2]. Numerous studies have explored the biological effects of RF-EMF exposure, including behavioral studies [1,3,4], reproductive system [5], metabolome [6,7,8], transcriptome [9,10], proteome [10,11,12], etc.

Evidence is increasing that RF-EMR may negatively affect human health. The brain, as the central nervous system that controls the body’s sensory functions, behavior, and mental symptoms, is sensitive to RF-EMF exposure [13], and lots of studies have explored the potential health hazards of RF-EMF with different frequencies to the brain. Previous studies suggested that exposure to RF-EMF in laboratory animals causes changes in their behavior, but there is no consensus regarding whether could cause detrimental effects [13]. For example, Zheng et al. [1] found that exposure to 2650 MHz-EMF had no obvious effect on spatial memory ability and did not cause depression-like behavior; however, anxiety-like behavior was induced in mice. In contrast, Wang et al. [12] suggested that long-term microwave exposure (2.856 and 9.375 GHz) could lead to different degrees of spatial learning and memory impairment.

In 2017, the Ministry of Industry and Information Technology of China issued a notice related to the use of the 3300–3600 MHz and 4800–5000 MHz frequency bands for the fifth-generation (5G) mobile communication system. In recent years, the number of 5G base stations and mobile phones in China has increased rapidly, and the opportunities and intensity of population exposure to RF radiation from 5G communication frequencies are gradually increasing. However, there are few studies on the biological effects were reported the biological effects of 5G communication signals in these frequency bands. Torres-Ruiz M et al. [14] found that early exposure of larval zebrafish embryos to a frequency of 3500 MHz for 4 h led to an increase in anxiety-like behaviors. Yang H et al. [4] found that acute exposure to 3.5 GHz RF electromagnetic radiation for 72 h did not cause anxiety-like behavior in guinea pigs, whereas Zhou et al. [15] found that 3.5 GHz radiofrequency radiation for 35 days could induce anxiety-like behaviors in mice. Moreover, our previous study found that 4.9 GHz radiofrequency radiation induced depression-like behavior in mice [16]. As one of the main frequency bands for 5G communication, it is necessary to evaluate the potential health hazards of 4.9 GHz exposure. Studies have reported that changes in peripheral energy metabolism might affect brain lipid levels and thereby cortical excitability [17], and a deregulated hippocampus proteome might influence the healthy functioning of the brain [11]. Therefore, in this study, we evaluated the effects of 4.9 GHz exposure on the metabolome of brain and serum and brain proteome using metabolomics and proteomics in mice.

## 2. Material and Methods

### 2.1. Animals

Male C57BL/6 mice aged 6–8 weeks (*n* = 12) were purchased from the Laboratory Animal Center of the Fourth Military Medical University (Xi’an, China) and maintained on a 12 h light/dark cycle at 25 °C room temperature in the animal cage. These mice were randomly divided into the Sham group (*n* = 6) and the 4.9 GHz radiofrequency exposure group (RF group, *n* = 6). Before the experiment, mice were housed for 1 week to adapt to the new environment. All animal experimental procedures were conducted in accordance with the Animal Welfare Committee of the Fourth Military Medical University (IACUC-20220123) and strictly based on the ARRIVE guidelines.

### 2.2. Radiofrequency Field Exposure

The 4.9 GHz radiofrequency exposure (pulse wave) system used in this study mainly includes a signal generator (Keysight N5171B, Keysight Technologies, Santa Rosa, CA, USA), a power amplifier (Bonn BLMA0860-100, BONN Elektronik, Holzkirchen, Germany), and a double ridged horn antenna (XJT DR10180, Sigten, Xi’an, China) [18]. Before the exposure experiment, the power density (50 ± 2.5 W/m^2^) was measured by an electromagnetic field meter (PMM8053A, PMM Costruzioni Electtroniche Centro Misure Radio Electriche S.r.l., Milan, Italy). During RF exposure, the mice were placed in a 32 mm × 42 mm × 80 mm box and exposure lasted for 1 h per day for 35 d. For the Sham group, the mice were placed in the same box every day, except that the irradiation device was not turned on. After 35 d of exposure, the brain tissue and serum of the mice were collected and stored at −80 °C for follow-up metabolomic (*n* = 6 per group) and proteomic (*n* = 3 per group) testing.

### 2.3. Metabolomic Analysis

The brain tissue and serum samples were sent to Majorbio Bio-Pharm Technology Co. Ltd. (Shanghai, China) for untargeted metabolomics testing. Briefly, metabolites of brain tissue and serum samples were extracted by adding extraction solutions (respectively, methanol: water = 4:1 (*v*:*v*), acetonitrile: methanol = 1:1 (*v*:*v*)) under low-temperature conditions, and then the supernatant metabolisms were used for liquid mass spectrometry detection by centrifugation. The instrument platform for this LC-MS analysis is the UHPLC-Q Exactive HF-X system of Thermo Fisher Scientific. After the mass spectrometry detection is completed, the raw data of LC/MS is preprocessed by Progenesis QI (Version QI2.4, Waters Corporation, Milford, CT, USA) software, and the metabolites were searched and identified by the metabolic public database (HMDB (https://hmdb.ca/ (accessed on 16 June 2022)), Metlin (https://metlin.scripps.edu/ (accessed on16 June 2022)) and Majorbio Database). These data, after the database search, are uploaded to the Majorbio cloud platform (https://cloud.majorbio.com (accessed on 16 June 2022)) for data analysis.

### 2.4. Proteomic Analysis

The brain tissue samples were sent to LC-Bio Technology Co., Ltd. (Hangzhou, China) for proteomics analysis. In this study, the Tandem Mass Tags (TMT) technique was used to quantify the proteome of mouse brain tissue in each group, which mainly included protein extraction and detection, enzyme digestion and salt removal, isotope labeling, and mass spectrometry detection. Based on the raw files detected by mass spectrometry, the protein identification quantitative analysis was carried out by searching the UniProt database and Proteome Discoverer.

### 2.5. Bioinformatics Analysis

When performing data analysis for metabolomics and proteomics, we first standardized and appropriately preprocessed the raw data (such as logarithmic transformations) to reduce the impact of abiotic variation, and the Shapiro–Wilk test results showed that nearly all of them fit a normal distribution. Based on the distribution characteristics of the pre-processed data and the fact that the sample size is large, we assume that the data approximately follows the normal distribution, and, accordingly, we use the student’s t-test to calculate the significance of the differences between the groups.

For metabolomic analysis, the differentially expressed metabolites (DEMs) were identified using the following screening criteria: VIP (variable importance in the projection) of ≥1 and *p* value < 0.05 (student’s *t*-test). Venn diagram was used to quickly understand the metabolite composition of different groups. Partial least-squares discriminant analysis (PLS-DA) was used to examine similarities in metabolite between samples and a volcano plot was used to show the difference in metabolites between groups. Cluster heat maps and VIP bar charts were used to visually demonstrate the importance and expression trends in DEMs between the two groups. DEMs between two groups were summarized, and mapped into their biochemical pathways through metabolic enrichment and pathway analysis based on an online KEGG database search (Kyoto Encyclopedia of Genes and Genomes, http://www.genome.jp/kegg (accessed on 16 June 2022)). KEGG pathway enrichment analyses were applied based on Fisher’s exact test.

For proteomics analysis, differentially expressed proteins (DEPs) were defined as proteins with a fold change greater than 1.2 and a *p* value (student’s *t*-test) less than 0.05. Principal component analysis (PCA) was used to reflect overall differences between groups and the variation between samples. Cluster heat maps and volcano plots were used to show the difference in protein expression between groups. Functional annotations for DEPs were carried out by GeneOntology (GO, www.geneontology.org (accessed on 26 September 2022)) and pathway analysis was performed using the KEGG database. GO enrichment and KEGG pathway enrichment analyses were applied based on Fisher’s exact test. The correlations between DEPs and DEMs were identified by Pearson correlation analysis. The value of the Pearson correlation coefficient ranges from −1 to 1, where 1 indicates a completely positive correlation, −1 indicates a completely negative correlation, and 0 indicates a wireless relationship. The greater the absolute value of the correlation coefficient, the stronger the correlation between the two samples.

## 3. Results

### 3.1. 4.9 GHz Radiofrequency Exposure Altered Metabolism Profile in Brain

**Figure 1:**
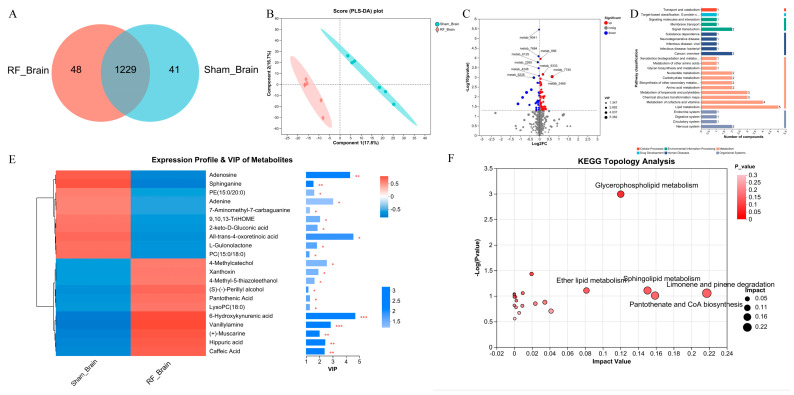
4.9 GHz RF exposure altered brain metabolism profile in mice. (**A**) Venn diagram metabolites detected between RF_Brain and Sham_Brain groups. (**B**) PLS_DA plot for metabolomics analysis of RF_Brain and Sham_Brain groups. (**C**) Volcano plot for DEMs between the RF_Brain and Sham_Brain groups. (**D**) KEGG function pathway annotation of DEMs between the RF_Brain and Sham_Brain groups. (**E**) VIP value analysis of DEMs annotated to KEGG pathway. * 0.01 < *p* ≤ 0.05, ** 0.001 < *p* ≤ 0.01, *** *p* ≤ 0.001 (**F**) KEGG topological analysis of brain DEMs.

A total of 1318 metabolites were identified by non-targeted metabolomics in mouse brain tissue, 1229 of which were shared by Sham_Brain and RF_Brain groups (Figure 1A). Initial examination of the total features, PLS-DA showed that there was a separation in metabolite composition of brain tissue between the RF_brain group and Sham_brain group (R^2^ = 0.9984, Q^2^ = 0.6637, Figure S1A), which shows that 17.8% and 16.7% of the variance in the original data is explained by Component1 and Component2, respectively (Figure 1B). Comparison of metabolite differences between groups (VIP > 1 and *p* value < 0.05), the contents of 74 DEMs were significantly up-regulated and 30 DEMs were significantly down-regulated in the RF_Brain group (Figure 1C), of which 21 DEMs were annotated to the KEGG functional pathway (Figure 1D). Variable importance in projection (VIP) analysis of the DEMs (Figure 1E) showed that 10 metabolites were significantly down-regulated including adenosine and sphinganine, and other 11 metabolites were significantly upregulated such as 6-Hydroxykynurenic acid and Vanillylamine in the RF group. KEGG enrichment analysis found (Figure 1F) that the DEMs in brain tissues were enriched in glycerophospholipid metabolism, sphingolipid metabolism, Limonene and pinene degradation, and Pantothenate and CoA biosynthesis. These results indicated that 35 d of RF exposure alters the metabolism profile in mouse brain tissue.

### 3.2. 4.9 GHz Radiofrequency Exposure Induced Disturbance of Metabolism in Serum

**Figure 2:**
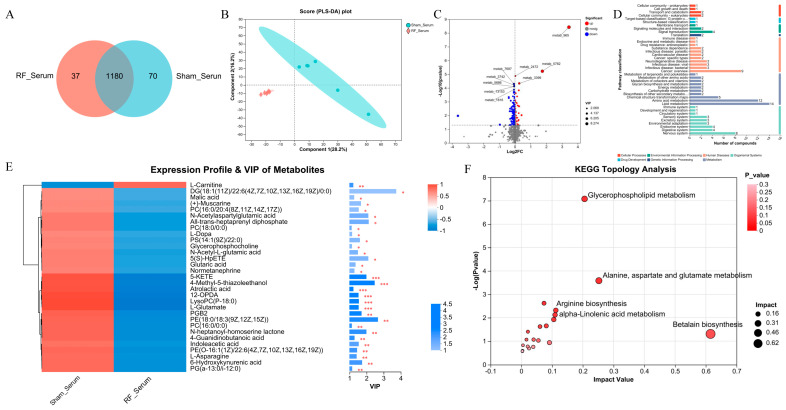
4.9 GHz RF exposure altered serum metabolism profile in mice. (**A**) Venn diagram metabolites detected between RF_Serum and Sham_Serum groups. (**B**) PLS_DA plot for metabolomics analysis of RF_Serum and Sham_Serum groups. (**C**) Volcano plot for DEMs between the RF_Serum and Sham_Serum groups. (**D**) KEGG function pathway annotation of DEMs between the RF_Serum and Sham_Serum groups. (**E**) VIP value analysis of DEMs annotated to KEGG pathway. * 0.01 < *p* ≤ 0.05, ** 0.001 < *p* ≤ 0.01, *** *p* ≤ 0.001 (**F**) KEGG topological analysis of brain DEMs.

To reveal the alterations in serum metabolites after 4.9 GHz RF exposure, we performed metabolic profiling using the serum from the RF_Serum and Sham_Serum groups. A total of 1287 metabolites were identified, and 1180 metabolites were shared by the RF_Serum and Sham_Serum groups (Figure 2A). Metabolites in serum were significantly different between the two groups by PLS-DA (R^2^ = 0.993, Q^2^ = 0.6166, Figure S1B), which shows that 28.2% and 16.2% of the variance in the original data is explained by Component1 and Component2, respectively (Figure 2B). The volcano plot showed that 153 DEMs were significantly up- or down-regulated in the RF_Serum group (Figure 2C), of which 31 DEMs were annotated to the KEGG functional pathway (Figure 2D). VIP analysis of the DEMs (Figure 2E) showed that only L-Carnitine was significantly down-regulated in the RF_Serum group, and other 30 metabolites such as 6-Hydroxykynurenic acid were significantly upregulated. The DEMs were enriched in glycerophospholipid metabolism, alanine, aspartate and glutamate metabolism, and betalain biosynthesis (Figure 2F). These results indicated that 35 d of RF exposure induced the disturbance of the metabolism profile in mouse serum tissue.

### 3.3. 4.9 GHz Radiofrequency Exposure Caused Deregulation of Proteins in Brain

**Figure 3:**
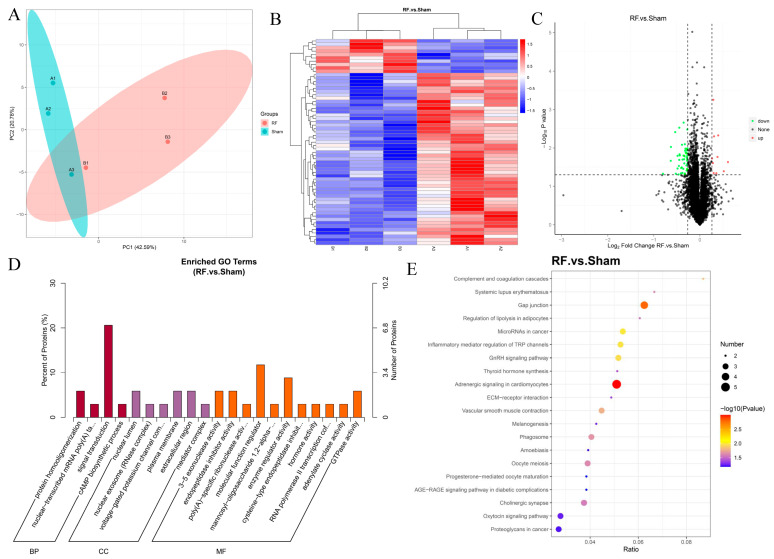
4.9 GHz RF exposure altered protein profile of the brain in mice. (**A**) PCA plot for proteomics analysis of RF and Sham groups. (**B**) Heatmap for DEPs in the comparison between the RF and Sham groups. (**C**) Volcano plot for DEPs between the RF and Sham groups. (**D**) GO enrichment analysis of the DEPs. (**E**) KEGG enrichment analysis of the DEPs.

A total of 7285 proteins were identified by TMT proteomics in mouse brain tissue, of which 7251 proteins could be quantified. The results of PCA showed that there were differences in proteome composition between the RF group and the Sham group (Figure 3A). Compared with the Sham group, a total of 61 DEPs were detected in the RF group, of which 10 were significantly up-regulated and 51 were significantly down-regulated (Figure 3B,C). GO enrichment analysis (Figure 3D) showed that the DEPs in brain tissues after RF were mainly located in the nuclear lumen, plasma membrane, and extracellular region, and played the role of molecular function regulator, enzyme regulator activity, 3–5 exonuclease activity, endopeptidase inhibitor activity, GTPase activity, and others. Additionally, these DEPs mainly participate in biological processes such as signal transduction, protein homooligomerization, nuclear-transcribed mRNA poly(A) tail shortening, and cAMP biosynthetic process. KEGG enrichment (Figure 3E) analysis showed that the DEPs were significantly enriched in gap junction and adrenergic signaling in cardiomyocytes. These results indicated that 35 d of RF exposure could significantly change the protein expression profile of mouse brain tissue.

### 3.4. Correlation Analysis of Metabolomics and Proteomics

**Figure 4:**
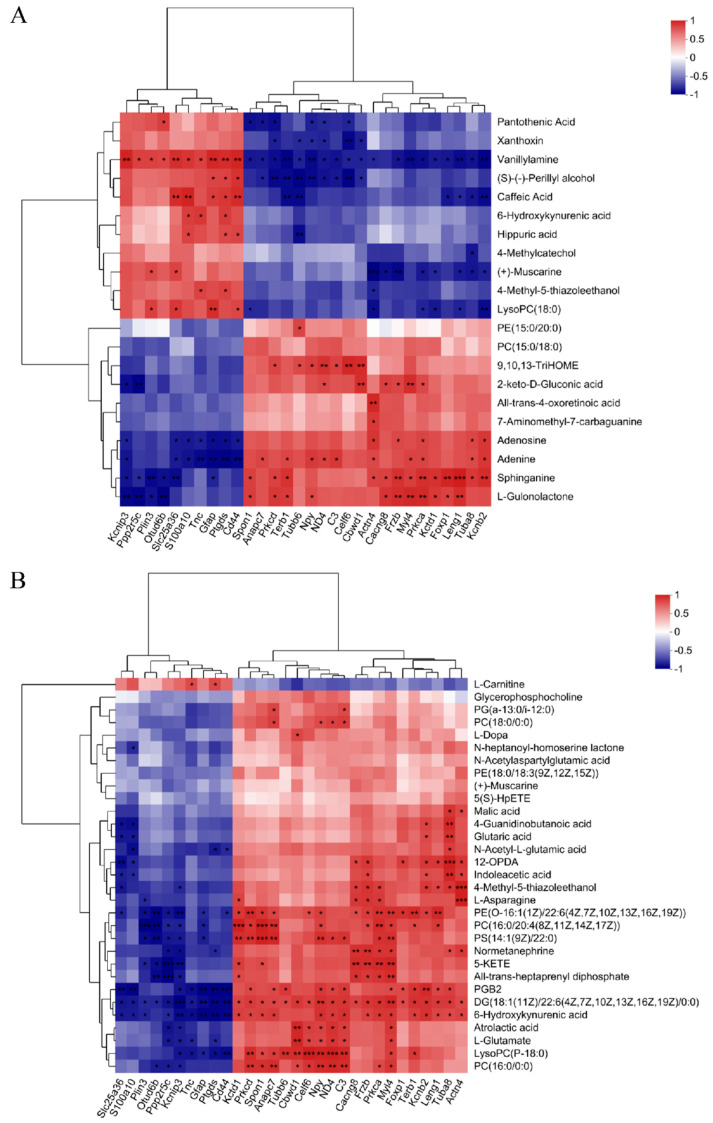
Integrated analysis of metabolomics and proteomics. (**A**) The correlation analysis of the DEMs and DEPs in the brain tissue. (**B**) The correlation analysis of the DEMs in serum and DEPs in the brain tissue. * 0.01 < *p* ≤ 0.05, ** 0.001 < *p* ≤ 0.01, *** *p* ≤ 0.001.

We integrated datasets of DEPs and DEMs to explore a global correlation between proteomics and metabolomics. As shown in Figure 4, the correlation coefficient and *p* value in the heatmap demonstrated the correlation between the DEMs and DEPs (*p* < 0.05), and most showed a significant correlation. In the correlation analysis results of DEMs and DEPs in brain tissue (Figure 4A, Table S1), 10 DEPs were positively correlated with 10 DEMs and negatively correlated with the other 11 DEMs. In particular, vanillylamine and sphinganine were significantly associated with most DEPs. The correlation (Figure 4B, Table S2) between DEMs of serum and DEPs of brain tissues showed 10 DEPs and 20 DEPs were negatively and positively correlated with all DEMs, except L-Carnitine. Among them, 13 DEMs such as PGB2 and 6-Hydroxykynurenic acid were significantly correlated with most DEPs.

## 4. Discussion

With the development of communication technology, people are inevitably affected by electromagnetic exposure. The available evidence focuses on the biological effects of RF-EMF exposure above 6 GHz [19,20,21], and there are few studies on the health hazards of 5G communication frequency under 6 GHz exposure. We aimed to determine the effects of RF-EMF 5G communication exposure on metabolomic and proteomic characterization in mice. It is widely believed that changes in peripheral energy metabolism affect neuronal activity in the brain, including the cerebral cortex [17]. However, until now, the molecular pathways that link changes in peripheral energy metabolism to brain function are still unknown. Therefore, we investigated the changes in the metabolism of brain tissue and serum. Notably, we found that the DEMs in the brain and serum were both enriched in glycerophospholipid metabolism. Compared with other tissues, lipids composition in the brain is abundant and diverse and plays an important role in neuronal function [22]. The lipid composition of the brain may influence perception and emotional behavior, which can lead to depression and anxiety disorders [22,23]. Previous clinical trials have found that depression is characterized by disturbances of peripheral and central lipid metabolism [24,25]. Wu et al. [26] revealed the dysfunction of glycerophospholipid metabolism in the hippocampus and prefrontal brain regions of late-onset depression rats. Zhang et al. [27] found that the disorder of glycerophospholipid metabolism in the hippocampus of the brain is a significant feature of depressed macaques in a nonhuman primate model of depression. In addition, Zhang et al. [28] found lipid metabolism disorder in the hippocampus and decreased expression of enzymes related to glycerophospholipid metabolism in the chronic unpredictable mild stress rat model of depression. Cogswell et al. [29] found that glycerophospholipid metabolism was correlated with the dim-light melatonin offset (DLMOff) biomarker model in the preliminary analysis for biomarkers of the circadian phase in humans based on plasma metabolomics. Similarly, in this study, we found that DEMs are significantly enriched in the glycerophospholipid metabolic pathway and are significantly down-regulated both in the brain and serum, which may also contribute to RF-EMF exposure-induced depression-like behavior in mice [16].

Proteins can be involved in transmembrane transport, energy metabolism, neuroprotection, or neurodegeneration, and are associated with various neuropathologies [11]. Verma et al. [30] and Sharma et al. [31] found that the protein expression in the brains of rats decreased after microwave radiation, which may be caused by excessive consumption or a reduction in protein. Consistent with this, we found 61 DEPs after 4.9 GHz exposure, of which 51 DEPs were significantly down-regulated. It is widely believed that protein synthesis occurs in neuronal dendrites and may be the cellular basis for learning and memory, and local protein synthesis and synaptic plasticity are closely related to the efficiency of communication between neurons [13]. Fragopoulou et al. [32] found that long-term exposure to microwave radiation induced the synthesis of proteins, including some proteins related to neuronal function, such as glial fibrillary acidic protein (GFAP), glial maturation factor (GMF), and cytoskeletal proteins, and some proteins related to metabolism in the brain. Belyaev et al. [33] found that exposure to 915 MHz microwaves in rats reduced 12 genes’ up- or down-regulation in cerebellar tissue, which codes for proteins with multiple functions, including neurotransmitter regulation, the blood–brain barrier, and melatonin production. Related to this, we found that the DEPs in brain tissue were enriched in gap junction in this study. Gap junctional communication is essential for many physiological events, including embryonic development, electrical coupling, metabolic transport, apoptosis, and tissue homeostasis. Previous studies have found that 3.5 GHz exposure increases the permeability of the blood–brain barrier in the cerebral cortex of mice. Therefore, we speculate that the decrease in DEPs (Tuba8, Tubb6, Prkca) enriched at the gap junction may be related to the change in blood–brain barrier permeability.

In the current study, we identified the correlations between DEMs and 30 DEPS and found that most of the DEMs were significantly related to DEPs. For example, the relative abundance of adenosine and sphinganine in the RF_Brain group was positively correlated with Tuba8 and Prkca (all *p* < 0.05). Recent studies have found that depression may be related to changes in sphingomyelin metabolism, in which changes in the expression abundance of sphingomyelin and ceramide can induce depressive or anxiety-like behavior in mice [34]. Due to their potential role in pathophysiology, sphingomyelin and ceramide are considered to be new therapeutic targets for depression [35,36]. The main component of intracellular sphingolipids is sphingomyelin, which can be hydrolyzed to ceramide by acid sphingomyelinase. Ceramide is the central lipid of the entire sphingolipid system, including various enzymes and molecules [37], and is involved in various balancing pathways, such as the N-acylation of sphinganine (as part of the synthesis of ceramide), and the degradation pathway of sphingomyelin and complex sphingolipids [38]. In animal experiments, sphingolipid metabolism disorder has been shown to be associated with hippocampal dysfunction in depression-like symptoms in mice [39]. Depression-like behavior induced by chronic unpredictable stress leads to increased levels of ceramide small molecules in the hippocampus and decreased levels of sphingomyelin small molecules in the hippocampus and frontal cortex of mice [40]. Sphingosine is a major component of sphingomyelin and ceramide synthesis, and in our study, changes in its abundance may induce depression-like behavior in mice by affecting the synthesis of sphingomyelin and ceramide. Determining whether the decrease in adenosine and sphinganine, which can be annotated in the sphingolipid signaling pathway and sphingolipid metabolism, is related to exposure-induced behavior change will require additional research. Tuba8 and Prkca are involved in gap junction, which can directly transfer small molecules, including ions, amino acids, nucleotides, second messengers, and other metabolites, between neighboring cells. Moreover, DEMs in serum such as 6-Hydroxykynurenic acid and indoleacetic acid were positively correlated with Actn4, Tuba8, and Kcnb2 (all *p* < 0.05). A decrease in the abundance of proteins (especially Tuba8) involved in gap junction may alter the BBB permeability of 6-Hydroxykynurenic acid, leading to a decreased expression of 6-Hydroxykynurenic acid in plasma. In addition, 6-Hydroxykynurenic acid has peroxynitrite scavenging activity [41] and an antagonistic effect on the N-methyl-D-aspartic acid (NMDA) receptor [42], suggesting that it may have neuroprotective effects. In addition, 6-HKA, as a derivative of kynurenic acid, can be involved in tryptophan metabolism, thereby inducing behavioral changes in mice.

A large number of epidemiological studies have shown that mobile phone radiation can cause neurobehavioral changes, including sleep disorders, depression, anxiety, and other neurological symptoms [43,44,45,46]. This study provides a scientific basis for evaluating potential health risks by exploring the effects of 5G radio frequency exposure on metabolome and proteome, which not only has guiding significance for the formulation of public health policies, but also provides a theoretical basis for the future security application of 5G technology. However, there are still some limitations in this study. On the one hand, the role of metabolite and protein changes in mediating the health hazards of electromagnetic radiation requires further investigation. On the other hand, considering the species differences, as well as the physiological and metabolic differences between mice and humans, caution should be exercised when extrapolating these results directly to humans.

## 5. Conclusions

In this study, we investigated the effects of 4.9 GHz exposure on the protein of the brain and the metabolism of brain tissue and serum in mice. The findings indicated that 4.9 GHz exposure altered metabolite expression patterns in brain tissue and serum, which may be related to glycerophospholipid metabolism. Moreover, proteomic analysis of serum suggested that 4.9 GHz induced protein profile imbalance and may alter gap junctional communication. These results revealed the biological effects of 5G communication frequency exposure, especially at the level of metabolism and protein regulation, and provided a new perspective and evidence for evaluating the safety of 5G communication technology.

## Data Availability

The data presented in this study are available on request from the corresponding author.

## Supplementary Materials

The following supporting information can be downloaded at: https://www.mdpi.com/article/10.3390/biology13100806/s1, Figure S1: The PLS-DA permutation test for the brain (A) and serum (B), respectively; Table S1: The correlation coefficient of the DEMs and DEPs in the brain tissue; Table S2: The correlation coefficient of the DEMs in serum and DEPs in the brain tissue.
