# Supplementary material for: Effects of 4.9 GHz Radiofrequency Field Exposure on Brain Metabolomic and Proteomic Characterization in Mice
Source: Biology (Basel). 2024 Oct 10;13(10):806. doi: 10.3390/biology13100806 (PMC11505847; doi:10.3390/biology13100806)
Supplement: Supplementary file 1 [file biology-13-00806-s001.zip › biology-3206745-supplementary.pdf]

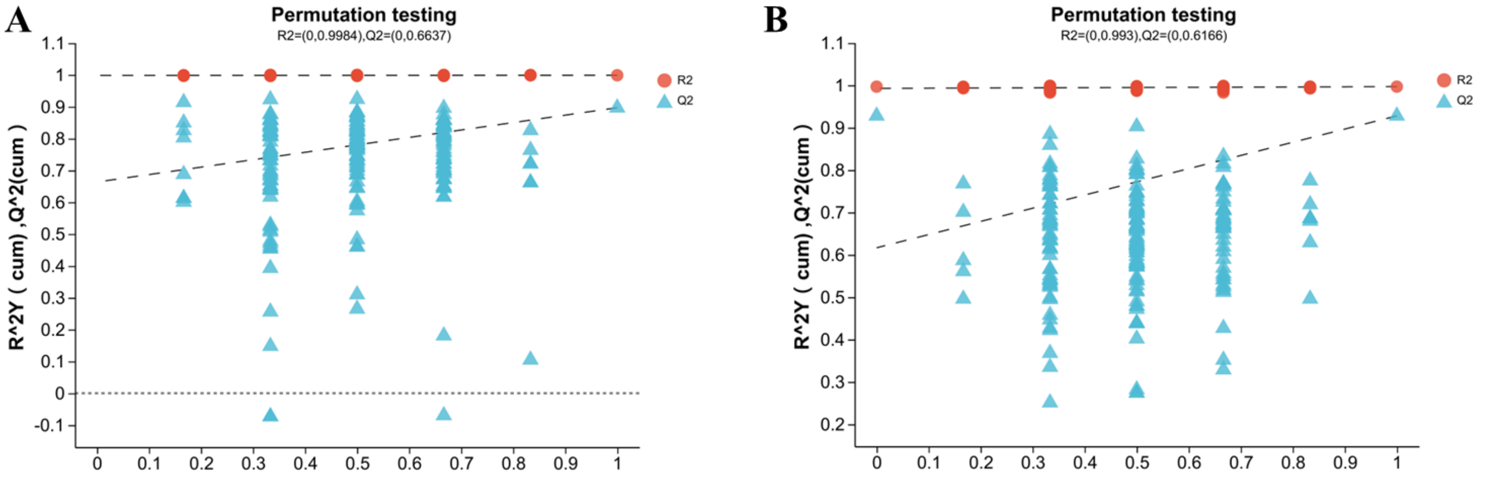

Figure S1 The PLS-DA permutation test for the brain (A) and serum (B), respectively.

Table S1 The correlation coefficient of the DEMs and DEPs in the brain tissue.

Metabolite	Kcnip3	Ppp2r5c	Plin3	Otud6b	Slc25a36	S100a10	Tnc	Gfap	Ptgds	Cd44	Spon1	Anapc7	Prkcd	Terb1	Tubb6	Npy	ND4	C3	Celf6	Cbwd1	Actn4	Cacng8	Frzb	Myl4	Prkca	Kctd1	Foxp1	Leng1	Tuba8	Kcnb2
Pantothenic Acid	0.7306	0.7539	0.7907	0.8636	0.4928	0.3479	0.5764	0.694	0.5864	0.6628	-0.9052	-0.8899	-0.9106	-0.7462	-0.6249	-0.8503	-0.8196	-0.8075	-0.8289	-0.6515	-0.348	-0.5265	-0.5756	-0.7939	-0.7425	-0.7813	-0.6048	-0.6673	-0.3603	-0.5733
Xanthoxin	0.7462	0.7024	0.5121	0.661	0.4982	0.5032	0.6226	0.5865	0.6625	0.7101	-0.6719	-0.6838	-0.8552	-0.7906	-0.816	-0.8175	-0.8324	-0.7608	-0.939	-0.8541	-0.1789	-0.4455	-0.4981	-0.7442	-0.5721	-0.4976	-0.5967	-0.5887	-0.4757	-0.5357
Vanillylamine	0.9686	0.8526	0.855	0.8312	0.9319	0.8967	0.8537	0.9556	0.9393	0.9655	-0.8755	-0.8686	-0.9063	-0.9538	-0.8476	-0.9301	-0.8757	-0.8908	-0.8387	-0.8371	-0.8188	-0.8046	-0.8843	-0.9289	-0.9129	-0.8668	-0.8767	-0.9201	-0.8925	-0.959
(S)-(-)-Perillyl alcohol	0.8015	0.6505	0.6549	0.6787	0.7424	0.7562	0.7967	0.8356	0.844	0.911	-0.801	-0.844	-0.931	-0.9244	-0.9638	-0.9258	-0.8914	-0.8762	-0.9602	-0.8126	-0.3867	-0.4442	-0.5572	-0.7609	-0.6552	-0.643	-0.7748	-0.7342	-0.6297	-0.775
Caffeic Acid	0.8112	0.591	0.6361	0.5794	0.934	0.9716	0.7816	0.872	0.8886	0.9428	-0.677	-0.702	-0.791	-0.927	-0.927	-0.809	-0.738	-0.749	-0.780	-0.735	-0.641	-0.5515	-0.674	-0.710	-0.671	-0.628	-0.881	-0.823	-0.879	-0.923

													3	3	6		6	8	2	7	1		7	9	7	2	9	3	7	
6-Hydroxykynurenic acid	0.5652	0.3473	0.2994	0.2071	0.6394	0.8615	0.8918	0.726	0.8772	0.8027	-0.4087	-0.5277	-0.4742	-0.5822	-0.7874	-0.6507	-0.674	-0.7075	-0.6098	-0.6528	-0.4818	-0.2861	-0.3819	-0.4705	-0.3944	-0.3447	-0.4306	-0.4108	-0.5955	-0.646
Hippuric acid	0.529	0.2602	0.3254	0.2395	0.6779	0.847	0.803	0.7391	0.8183	0.8364	-0.4758	-0.5923	-0.6061	-0.7082	-0.9188	-0.6973	-0.6753	-0.6974	-0.7181	-0.6042	-0.2981	-0.1288	-0.2737	-0.4214	-0.3302	-0.3304	-0.5816	-0.4766	-0.5536	-0.6766
4-Methylcatechol	0.6692	0.6101	0.4272	0.4345	0.6955	0.6982	0.3947	0.4555	0.5447	0.5125	-0.2834	-0.2088	-0.341	-0.5354	-0.3714	-0.3603	-0.3257	-0.3103	-0.2925	-0.5498	-0.7787	-0.7893	-0.7853	-0.5926	-0.6086	-0.4419	-0.5922	-0.6434	-0.8804	-0.6515
(+)-Muscarine	0.7844	0.7412	0.8143	0.7408	0.8463	0.7036	0.4865	0.7236	0.6221	0.6465	-0.6584	-0.5691	-0.5766	-0.6825	-0.3915	-0.5629	-0.4664	-0.5086	-0.3683	-0.4793	-0.9899	-0.9072	-0.9344	-0.7552	-0.8715	-0.8251	-0.7731	-0.8641	-0.8848	-0.8458
4-Methyl-5-thiazoleethanol	0.7685	0.7125	0.6761	0.6066	0.686	0.7252	0.8445	0.8102	0.8441	0.7621	-0.678	-0.7053	-0.5899	-0.604	-0.5501	-0.7278	-0.7319	-0.7819	-0.5563	-0.6687	-0.8372	-0.7244	-0.7528	-0.7587	-0.7785	-0.7363	-0.4973	-0.6244	-0.6759	-0.7322
LysoPC(18:0)	0.7671	0.6364	0.8595	0.7105	0.8931	0.8111	0.7335	0.9311	0.8005	0.8324	-0.8137	-0.809	-0.7091	-0.7684	-0.6144	-0.7504	-0.6539	-0.735	-0.5383	-0.4995	-0.9104	-0.6944	-0.7857	-0.7319	-0.8355	-0.8779	-0.7851	-0.8377	-0.7814	-0.9211
PE(15:0/20:0)	-0.3428	-0.0833	-0.0495	-0.0235	-0.462	-0.682	-0.627	-0.4873	-0.6358	-0.6522	0.2332	0.3586	0.4453	0.5527	0.8416	0.5196	0.5279	0.5122	0.6403	0.5335	-0.0021	-0.0868	0.0438	0.2271	0.0743	0.0429	0.4073	0.2597	0.3795	0.4433
PC(15:0/18:0)	-0.627	-0.7203	-0.6618	-0.6959	-0.2976	-0.2494	-0.6761	-0.6169	-0.5936	-0.5307	0.7564	0.7787	0.6343	0.4364	0.368	0.7196	0.7645	0.787	0.6021	0.5908	0.4706	0.5575	0.5442	0.7225	0.6983	0.7155	0.238	0.4155	0.2208	0.4036
9,10,13-TriHOME	-0.7877	-0.7612	-0.5024	-0.6497	-0.4734	-0.5351	-0.7604	-0.6289	-0.7606	-0.7488	0.6803	0.7174	0.8348	0.7508	0.8193	0.8642	0.9175	0.8528	0.9619	0.9395	0.2451	0.4966	0.5311	0.796	0.6092	0.5152	0.5013	0.5388	0.4706	0.5261
2-keto-D-Gluconic acid	-0.9117	-0.961	-0.6557	-0.7961	-0.5782	-0.5612	-0.6868	-0.631	-0.7385	-0.6951	0.6913	0.6524	0.7626	0.7221	0.6096	0.7874	0.8237	0.7698	0.7866	0.9262	0.6052	0.8583	0.8381	0.9331	0.8288	0.6836	0.5598	0.6858	0.6828	0.6208
All-trans-4-oxoretinoic acid	-0.6319	-0.5957	-0.696	-0.5708	-0.6989	-0.6187	-0.526	-0.674	-0.592	-0.557	0.5588	0.5144	0.4014	0.4792	0.2619	0.4686	0.4048	0.4795	0.2238	0.3563	0.9578	0.7714	0.7877	0.6161	0.7526	0.7366	0.5384	0.6616	0.7107	0.715

			8				1	1	1																					
7-Aminomethyl-7-carbaguanine	-0.6652	-0.6486	-0.551	-0.4943	-0.6212	-0.6326	-0.5919	-0.5945	-0.6428	-0.5507	0.446	0.4176	0.3558	0.4419	0.3018	0.466	0.4606	0.4985	0.2871	0.5196	0.8855	0.792	0.7785	0.6429	0.7015	0.6065	0.4288	0.5672	0.7192	0.6325
Adenosine	-0.8476	-0.7311	-0.7699	-0.6778	-0.8661	-0.8732	-0.8551	-0.914	-0.9062	-0.8779	0.7545	0.7679	0.7031	0.7704	0.6857	0.7964	0.7548	0.8066	0.6308	0.6932	0.9034	0.7612	0.8268	0.8061	0.8403	0.8079	0.7125	0.7892	0.8326	0.8936
Adenine	-0.8844	-0.7573	-0.7701	-0.7014	-0.8641	-0.8935	-0.9271	-0.9511	-0.9648	-0.9406	0.8042	0.836	0.7874	0.8314	0.7927	0.8826	0.8536	0.8937	0.7533	0.7812	0.8285	0.723	0.7993	0.8443	0.8389	0.8061	0.7277	0.7913	0.8117	0.8996
Sphinganine	-0.8883	-0.8142	-0.9323	-0.9028	-0.9247	-0.7397	-0.5415	-0.8356	-0.6917	-0.7931	0.8562	0.7723	0.8526	0.9121	0.638	0.7682	0.6488	0.6668	0.6486	0.6086	0.8422	0.8502	0.9208	0.8623	0.9259	0.9114	0.9646	0.9978	0.8859	0.9352
L-Gulonolactone	-0.9405	-0.9519	-0.8956	-0.9679	-0.775	-0.6041	-0.5554	-0.7492	-0.6757	-0.7423	0.8646	0.7756	0.8936	0.8773	0.6176	0.8141	0.7501	0.7271	0.7534	0.7597	0.7295	0.9063	0.9323	0.9547	0.9478	0.8849	0.8436	0.9246	0.7901	0.8071

Table S2 The correlation coefficient of the DEMs in serum and DEPs in the brain tissue.

Metabolite	Slc25a36	S100a10	Plin3	Otud6b	Ppp2r5c	Kcnip3	Tnc	Gfap	Ptgds	Cd44	Kctd1	Prkcd	Spon1	Anapc7	Tubb6	Cbwd1	Celf6	Npy	ND4	C3	Cacng8	Frzb	Prkca	Myl4	Foxp1	Terb1	Kcnb2	Leng1	Tuba8	Actn4
L-Carnitine	0.566	0.7677	0.2943	0.3117	0.5792	0.6802	0.8126	0.5916	0.8234	0.6998	-0.3521	-0.4334	-0.3478	-0.4071	-0.645	-0.798	-0.5848	-0.6	-0.6656	-0.652	-0.5559	-0.5736	-0.507	-0.6148	-0.3647	-0.531	-0.5704	-0.4252	-0.6745	-0.5669
Glycerophosphocholine	-0.0613	-0.0799	-0.2961	-0.4642	-0.4756	-0.4288	-0.4665	-0.3301	-0.3966	-0.4089	0.2971	0.6422	0.5282	0.5788	0.5399	0.622	0.7686	0.6352	0.7046	0.6396	0.1258	0.1434	0.3087	0.4952	0.1726	0.4368	0.1396	0.2015	-0.0196	-0.1703
PG(a-13:0/i-12:0)	-0.3575	-0.3861	-0.5584	-0.4843	-0.4135	-0.4723	-0.7741	-0.72	-0.6639	-0.6406	0.5996	0.6306	0.7304	0.8337	0.5624	0.4626	0.6347	0.7433	0.7578	0.8196	0.2029	0.2772	0.494	0.5156	0.2746	0.4735	0.4537	0.3423	0.1396	0.3126
PC(18:0/0:0)	-0.3286	-0.3223	-0.6244	-0.6451	-0.6252	-0.6048	-0.7614	-0.6863	-0.6651	-0.6337	0.6651	0.7285	0.7955	0.8616	0.5587	0.6254	0.7431	0.8114	0.8504	0.8721	0.3791	0.4136	0.6146	0.6786	0.2935	0.5286	0.4349	0.4054	0.1787	0.3208
L-Dopa	-0.3738	-0.4085	-0.3057	-0.5206	-0.6761	-0.6709	-0.4336	-0.3383	-0.5066	-0.5177	0.293	0.6418	0.4066	0.3814	0.6388	0.8327	0.7693	0.5975	0.6419	0.5312	0.4918	0.4914	0.4594	0.6574	0.4671	0.6332	0.3802	0.4719	0.4918	0.1294
N-heptanoyl-homoserine	-0.6255	-0.8119	-0.2805	-0.2525	-0.4977	-0.6302	-0.7369	-0.5766	-0.7778	-0.6694	0.3316	0.3527	0.2821	0.3278	0.5902	0.6864	0.4629	0.5081	0.5434	0.5459	0.5507	0.5789	0.4782	0.5412	0.4103	0.5082	0.6094	0.4499	0.7381	0.6373

lactone																														
N-Acetylaspartylglutamic acid	-0.3652	-0.58	-0.1712	-0.2068	-0.5132	-0.5504	-0.7132	-0.4296	-0.6868	-0.5209	0.2445	0.2639	0.2172	0.2765	0.4443	0.7003	0.4343	0.4568	0.5566	0.5412	0.5005	0.4779	0.4075	0.5135	0.1416	0.3236	0.3793	0.2442	0.5061	0.4868
PE(18:0/18:3(9Z,12Z,15Z))	-0.3656	-0.3334	-0.586	-0.5257	-0.597	-0.53	-0.5774	-0.5551	-0.5298	-0.4268	0.6561	0.3539	0.5501	0.5503	0.1509	0.3902	0.2648	0.4819	0.5028	0.5648	0.6361	0.6069	0.6594	0.5868	0.1946	0.2766	0.4352	0.3897	0.3574	0.72
(+)-Muscarine	-0.2869	-0.3592	-0.2833	-0.3687	-0.6516	-0.5617	-0.4613	-0.2897	-0.4738	-0.3247	0.3487	0.2133	0.2282	0.1977	0.1601	0.5975	0.2705	0.3326	0.4134	0.3856	0.7146	0.6316	0.5277	0.5753	0.1281	0.2352	0.3053	0.3014	0.4946	0.5873
5(S)-HpETE	-0.5207	-0.6597	-0.2187	-0.1278	-0.3391	-0.4393	-0.5286	-0.4334	-0.5697	-0.452	0.2754	0.1041	0.1323	0.1523	0.2858	0.3948	0.134	0.2608	0.2742	0.3113	0.5111	0.5163	0.3895	0.3606	0.2723	0.2782	0.4962	0.3385	0.6404	0.6977
Malic acid	-0.7772	-0.7826	-0.444	-0.3743	-0.4909	-0.6153	-0.4166	-0.5298	-0.5697	-0.5589	0.4542	0.3221	0.292	0.2364	0.4059	0.4432	0.2417	0.3492	0.2818	0.2992	0.7017	0.7349	0.5729	0.5145	0.6498	0.5524	0.7253	0.6652	0.9013	0.8205
4-Guanidinobutanoic acid	-0.897	-0.9113	-0.5139	-0.4356	-0.499	-0.6905	-0.5333	-0.6707	-0.693	-0.7257	0.508	0.502	0.4206	0.3903	0.637	0.5369	0.4375	0.51	0.4231	0.4388	0.637	0.7185	0.5991	0.5701	0.8057	0.7332	0.8466	0.7662	0.9534	0.7675
Glutaric acid	-0.8779	-0.874	-0.4891	-0.3801	-0.4126	-0.6111	-0.4534	-0.6298	-0.6185	-0.6618	0.4794	0.4258	0.3698	0.3337	0.5569	0.4181	0.3302	0.4255	0.3216	0.3513	0.5883	0.6735	0.5493	0.4869	0.7846	0.6717	0.8209	0.7363	0.9213	0.7679
N-Acetyl-L-glutamic acid	-0.8096	-0.9136	-0.5274	-0.4736	-0.6384	-0.7821	-0.806	-0.7628	-0.8754	-0.8137	0.5669	0.5505	0.5106	0.5335	0.6906	0.735	0.5755	0.6663	0.6568	0.6758	0.6937	0.7459	0.677	0.6998	0.6281	0.6948	0.8032	0.6711	0.8701	0.7999
12-OPDA	-0.9199	-0.8337	-0.7077	-0.6605	-0.6929	-0.8051	-0.4984	-0.7104	-0.6714	-0.7179	0.7006	0.6083	0.5773	0.4989	0.561	0.5717	0.4652	0.5762	0.477	0.4901	0.8381	0.8917	0.7877	0.728	0.8707	0.7835	0.8894	0.89	0.9858	0.891
Indoleacetic acid	-0.8527	-0.8209	-0.5826	-0.5469	-0.6462	-0.7543	-0.4877	-0.6273	-0.651	-0.6606	0.5865	0.496	0.4496	0.3812	0.5124	0.5739	0.4014	0.4964	0.4241	0.4288	0.8097	0.8461	0.7081	0.668	0.766	0.6932	0.8135	0.7916	0.9659	0.8592
4-Methyl-5-thiazoleethanol	-0.8743	-0.8094	-0.7844	-0.6964	-0.7377	-0.8195	-0.6604	-0.811	-0.7631	-0.7552	0.8106	0.6109	0.6797	0.6356	0.5188	0.5796	0.4662	0.6543	0.5833	0.6315	0.8633	0.9055	0.861	0.7776	0.7481	0.7173	0.8813	0.8349	0.8953	0.9865
L-Asparagine	-0.754	-0.6335	-0.8174	-0.7407	-0.7591	-0.7672	-0.5664	-0.7374	-0.6478	-0.6321	0.8496	0.5608	0.693	0.6281	0.3467	0.4902	0.3728	0.5943	0.5292	0.5835	0.8901	0.9016	0.88	0.7677	0.6428	0.6064	0.7803	0.7786	0.7724	0.9815
PE(O-16:1(1Z)/22:6(4Z,7Z,10Z,13Z,16Z,19Z))	-0.8377	-0.6777	-0.9074	-0.9478	-0.8851	-0.9301	-0.6037	-0.8211	-0.7246	-0.8188	0.887	0.9511	0.9052	0.8384	0.728	0.7478	0.8133	0.8664	0.7836	0.7711	0.8134	0.8758	0.9178	0.9258	0.9154	0.9474	0.8669	0.9534	0.7973	0.6905
PC(16:0/20:4(8Z,11Z,14Z,17Z	-0.7472	-0.5481	-0.9861	-0.9652	-0.836	-0.8435	-0.6158	-0.8555	-0.6783	-0.7567	0.9836	0.9046	0.9843	0.9328	0.5817	0.594	0.7073	0.852	0.767	0.8	0.7661	0.8232	0.9374	0.8821	0.7905	0.825	0.8106	0.8794	0.6286	0.7347

))																														
PS(14:1(9Z)/22:0)	-0.6621	-0.5389	-0.9144	-0.9315	-0.8767	-0.8675	-0.7461	-0.855	-0.7607	-0.7892	0.9316	0.9181	0.9761	0.9611	0.6435	0.7266	0.8088	0.9196	0.8876	0.9059	0.7361	0.7789	0.9123	0.9181	0.6714	0.7983	0.7427	0.7836	0.559	0.6568
Normetanephrine	-0.7439	-0.7219	-0.7519	-0.7673	-0.9	-0.9079	-0.7574	-0.7715	-0.8177	-0.7626	0.7941	0.6896	0.7164	0.684	0.5656	0.7962	0.6357	0.7586	0.7549	0.7613	0.9197	0.9226	0.8978	0.9034	0.6295	0.7153	0.7774	0.7653	0.8134	0.8768
5-KETE	-0.6531	-0.5448	-0.8375	-0.9127	-0.9872	-0.9236	-0.6571	-0.7227	-0.7152	-0.6968	0.8649	0.7896	0.8192	0.7563	0.5148	0.7973	0.6984	0.796	0.7886	0.7752	0.9421	0.9284	0.9491	0.9649	0.6323	0.7312	0.7079	0.7871	0.7022	0.7757
All-trans-heptaprenyl diphosphate	-0.5527	-0.4055	-0.8052	-0.9265	-0.9813	-0.8761	-0.5299	-0.6145	-0.5937	-0.5964	0.8201	0.7805	0.7906	0.7065	0.4454	0.7612	0.6831	0.7438	0.738	0.7038	0.9088	0.8801	0.9063	0.9367	0.5936	0.6898	0.6096	0.7465	0.6118	0.6564
PGB2	-0.9247	-0.9586	-0.7493	-0.6775	-0.7101	-0.883	-0.9107	-0.964	-0.974	-0.9821	0.769	0.8234	0.7919	0.8259	0.8825	0.7856	0.796	0.8925	0.8445	0.8774	0.6678	0.7692	0.8004	0.8178	0.8166	0.9025	0.9446	0.8316	0.8598	0.7795
DG(18:1(11Z)/22:6(4Z,7Z,10Z,13Z,16Z,19Z)/0:0)	-0.884	-0.8716	-0.8177	-0.821	-0.8917	-0.9814	-0.8693	-0.9187	-0.944	-0.9424	0.8391	0.8791	0.8425	0.8361	0.8221	0.8871	0.8411	0.9215	0.8916	0.8956	0.838	0.8974	0.9131	0.9495	0.8132	0.9168	0.9144	0.8794	0.8804	0.8129
6-Hydroxykynurenic acid	-0.8668	-0.8641	-0.8263	-0.7765	-0.819	-0.9226	-0.9125	-0.9594	-0.9557	-0.9421	0.8585	0.8382	0.8578	0.8752	0.7881	0.8045	0.7856	0.9135	0.8811	0.9151	0.7727	0.8425	0.8908	0.896	0.7563	0.8608	0.9085	0.8313	0.8147	0.8379
Atrolactic acid	-0.493	-0.5411	-0.5849	-0.7184	-0.8909	-0.8591	-0.8004	-0.654	-0.7942	-0.7264	0.6253	0.7698	0.6974	0.7115	0.6792	0.9545	0.8591	0.8424	0.9102	0.8624	0.709	0.7015	0.7464	0.8878	0.4481	0.6848	0.5548	0.5688	0.5481	0.4796
L-Glutamate	-0.5115	-0.5871	-0.5557	-0.6743	-0.8703	-0.8547	-0.8308	-0.6626	-0.8231	-0.74	0.6023	0.7338	0.6625	0.6852	0.6855	0.9575	0.8365	0.828	0.9002	0.8575	0.7084	0.7022	0.7322	0.8717	0.4357	0.6714	0.5678	0.5555	0.5774	0.5115
LysoPC(P-18:0)	-0.7002	-0.7381	-0.6784	-0.7386	-0.7898	-0.8837	-0.8867	-0.8441	-0.9074	-0.9192	0.6904	0.9361	0.8281	0.8708	0.9305	0.932	0.9956	0.9736	0.9804	0.9564	0.5748	0.6505	0.7417	0.8681	0.6913	0.8944	0.7501	0.7152	0.6377	0.4616
PC(16:0/0:0)	-0.5593	-0.5007	-0.7889	-0.86	-0.859	-0.8458	-0.785	-0.7899	-0.7731	-0.7808	0.8093	0.9188	0.9146	0.9266	0.7102	0.8224	0.9	0.9386	0.9476	0.9374	0.6417	0.6782	0.8193	0.8974	0.5773	0.7757	0.6439	0.6775	0.4744	0.4863
